# Effectiveness of an Electronic Communication Tool on Transitions in Care From the Intensive Care Unit: Protocol for a Cluster-Specific Pre-Post Trial

**DOI:** 10.2196/18675

**Published:** 2021-01-08

**Authors:** Jeanna Parsons Leigh, Rebecca Brundin-Mather, Liam Whalen-Browne, Devika Kashyap, Khara Sauro, Andrea Soo, Jennie Petersen, Monica Taljaard, Henry T Stelfox

**Affiliations:** 1 School of Health Administration Faculty of Health Dalhousie University Halifax, NS Canada; 2 Department of Critical Care Medicine Cumming School of Medicine University of Calgary Calgary, AB Canada; 3 Critical Care Medicine Alberta Health Services Calgary, AB Canada; 4 Department of Community Health Sciences Cumming School of Medicine University of Calgary Calgary, AB Canada; 5 O'Brien Institute for Public Health Cumming School of Medicine University of Calgary Calgary, AB Canada; 6 Department of Surgery Cumming School of Medicine University of Calgary Calgary, AB Canada; 7 Department of Oncology Tom Baker Cancer Centre Calgary, AB Canada; 8 Arnie Charbonneau Cancer Institute Health Research Innovation Centre University of Calgary Calgary, AB Canada; 9 Faculty of Applied Health Sciences Brock University St Catharines, ON Canada; 10 Clinical Epidemiology Program Ottawa Hospital Research Institute Ottawa, ON Canada; 11 School of Epidemiology and Public Health University of Ottawa Ottawa, ON Canada

**Keywords:** patient transfers, interprovider communication, transitions in care, electronic charting, clinical documentation, discharge tools, patient discharge summaries, electronic transfer summaries, intensive care unit, electronic tool, ICU, protocol, effective, communication, transfer, patient, transition

## Abstract

**Background:**

Transitions in care are vulnerable periods in health care that can expose patients to preventable errors due to incomplete or delayed communication between health care providers. Transitioning critically ill patients from intensive care units (ICUs) to other patient care units (PCUs) is particularly risky, due to the high acuity of the patients and the diversity of health care providers involved in their care. Instituting structured documentation to standardize written communication between health care providers during transitions has been identified as a promising means to reduce communication breakdowns. We developed an evidence-informed, computer-enabled, ICU-specific structured tool—an electronic transfer (e-transfer) tool—to facilitate and standardize the composition of written transfer summaries in the ICUs of one Canadian city. The tool consisted of 10 primary sections with a user interface combination of structured, automated, and free-text fields.

**Objective:**

Our overarching goal is to evaluate whether implementation of our e-transfer tool will improve the completeness and timeliness of transfer summaries and streamline communications between health care providers during high-risk transitions.

**Methods:**

This study is a cluster-specific pre-post trial, with randomized and staggered implementation of the e-transfer tool in four hospitals in Calgary, Alberta. Hospitals (ie, clusters) were allocated randomly to cross over every 2 months from control (ie, dictation only) to intervention (ie, e-transfer tool). Implementation at each site was facilitated with user education, point-of-care support, and audit and feedback. We will compare transfer summaries randomly sampled over 6 months postimplementation to summaries randomly sampled over 6 months preimplementation. The primary outcome will be a binary composite measure of the timeliness and completeness of transfer summaries. Secondary measures will include overall completeness, timeliness, and provider ratings of transfer summaries; hospital and ICU lengths of stay; and post-ICU patient outcomes, including ICU readmission, adverse events, cardiac arrest, rapid response team activation, and mortality. We will use descriptive statistics (ie, medians and means) to describe demographic characteristics. The primary outcome will be compared within each hospital pre- and postimplementation using separate logistic regression models for each hospital, with adjustment for patient characteristics.

**Results:**

Participating hospitals were cluster randomized to the intervention between July 2018 and January 2019. Preliminary extraction of ICU patient admission lists was completed in September 2019. We anticipate that evaluation data collection will be completed by early 2021, with first results ready for publication in spring or summer 2021.

**Conclusions:**

This study will report the impact of implementing an evidence-informed, computer-enabled, ICU-specific structured transfer tool on communication and preventable medical errors among patients transferred from the ICU to other hospital care units.

**Trial Registration:**

ClinicalTrials.gov NCT03590002; https://www.clinicaltrials.gov/ct2/show/NCT03590002

**International Registered Report Identifier (IRRID):**

DERR1-10.2196/18675

## Introduction

### Background

Complete and timely communication between health care providers is integral to seamless transitions in care [1-3]. The transfer of critically ill patients from the intensive care unit (ICU) to another patient care unit (PCU) is a particularly vulnerable period in patient care, due to the high acuity of patients [4-6] as well as the number of health care providers involved and their professional diversity [7-10]. The movement of patients between units requires a high degree of collaboration, with verbal and written communication between health care providers [1-3] as well as patients and families [1,11-13]. Suboptimal communication during transitions can have profound implications for patients, families, and the health care system [14-16], including increased risk of preventable medical errors, adverse events, redundant testing, readmissions, and dissatisfaction with the quality of care [17-24].

An ICU transfer summary is a clinical document that ICU physicians and nurse practitioners (NPs) often prepare to describe a patient’s stay in the ICU, including active and resolved health issues and the current care plan. The transfer summary is intended to support verbal communication between transferring and accepting medical teams and should provide sufficient detail to serve as a stand-alone communication [25]. Complete and timely exchanges of patient care information during transitions in care are critical, not only for immediate continuity of care but also for efficient coordination of future care [26,27]. As such, the transfer summary should be easily accessible to inpatient and outpatient health care providers as part of the patient’s permanent health care record.

Standardized transfer protocols that structure documentation are integral for preventing failures in patient care due to incomplete and delayed exchange of information [21,28-30]. However, their value can be limited by the very methods used to produce the document. While quick for the clinician to prepare, traditional methods like dictation or handwritten notes in the patient chart have been associated with inaccurate, incomplete, and lengthy delays in communication [17,20,31,32], particularly in comparison to transfer summaries prepared using electronic standardized tools [26,33-39]. The advancements of clinical information systems (CISs) and integrated electronic medical records (EMRs) provide a prime opportunity to optimize text-based communication. Structured templates can facilitate completeness of important patient information as well as substantiate and prompt verbal communication between health care providers at the point of care. They can also provide flexibility, permitting physicians to create a “living” document that can be edited over the course of stay and finalized at the point of patient transfer, effectively facilitating clinical workflow in complex settings. Despite the potential for optimizing efficient interprovider communication, the use of standardized tools to prepare ICU transfer summaries has not been widespread, with factors such as usability [39], cost, and workload [40] being barriers to adoption.

### Local Initiative to Standardize Transfer Summaries: The Electronic ICU Transfer Tool

In 2017, we began designing an evidence-informed, computer-enabled, ICU-specific communication tool in the primary, integrated patient care CIS—Sunrise Clinical Manager (Eclipsys Corporation)—used in four acute care hospitals in a single Canadian city. This work was initiated as a quality improvement project to improve upon the conventional system of dictation that physicians and NPs—herein called ICU clinicians—use to prepare medical transfer summaries for ICU patients [41]. To dictate a summary, ICU clinicians use eScription, 2010 release (Nuance Communications), a health information management dictation, speech recognition, and transcription (DST) platform. The clinician verbalizes relevant patient transfer information to create a voice file that is run through speech recognition software to create a text report. The report is then edited by a transcriptionist and sent to the designated ICU clinician for approval before being uploaded for electronic viewing in the CIS as well as to a provincial, web-based health data repository accessible by community-based physicians (Alberta Netcare).

**Figure 1:**
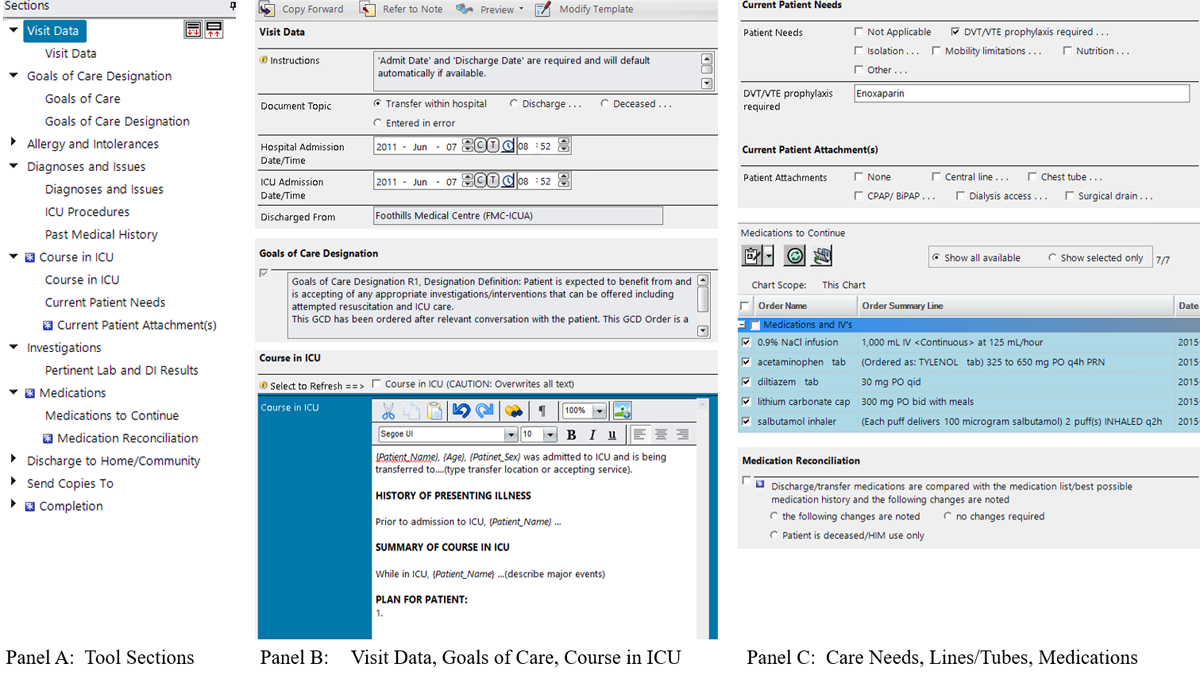
Electronic transfer tool sections and screenshots. ICU: intensive care unit.

The content and structure of the ICU electronic transfer (e-transfer) tool was based on a national survey of existing transfer summary tools [42], subsequent consensus-based recommendations of two independent multidisciplinary groups of health care providers [41,43], and a heuristic evaluation [41]. The e-transfer tool consists of 10 overarching document sections: visit data, goals of care, allergy and intolerances, diagnoses and visit issues, course in ICU, investigations, medications, discharge to home or community, send copies to, and completion. These sections are designed with a user interface combination of structured fields (eg, checkboxes); automated fields, which pull in relevant patient data from other CIS locations; and free-text fields (see Figure 1). The tool permits ICU clinicians to open an ICU summary as a clinical document directly in the patient’s EMR and edit the summary over the course of the patient’s ICU stay. As with the DST system, the designated ICU clinician must approve transfer summaries. The summaries remain in the CIS and are uploaded to the provincial repository.

In a small pilot test of the e-transfer tool in one ICU [42], electronic summaries had a significantly greater proportion of essential information fields completed overall (median 87.5%) than those prepared by dictation (median 62.5%) and were available to receiving teams closer to patient release (2.3 versus 45.0 hours). Primary users of the e-transfer tool also responded positively to its use, establishing favorable evidence to scale up implementation across additional municipal hospitals.

### Objective

In this study, we will evaluate the effectiveness of the ICU e-transfer tool for improved completeness and timeliness of transfer summaries and reduced adverse patient outcomes by comparing transfer summaries produced postimplementation to those produced preimplementation.

### Conceptual Framework

We will apply the Donabedian three-pronged model of health care quality (ie, structure, process, and outcome) [44] and the National Health Service Sustainability Model [45] to frame our evaluation of the e-transfer tool. The Donabedian model has been successfully used in multiple contexts to support quality improvement initiatives related to structures (ie, health care context), processes (ie, actions and events in health care), patient outcomes (ie, effects on health status, quality, knowledge, or behavior), and use of resources [46,47]. Similarly, the National Health Service Sustainability Model has been successfully used to predict the likelihood of sustainability for improvement initiatives [48]. In drawing from each of these models, we will ensure that we identify areas that need strengthening and that we are well positioned for sustainability and continual improvement.

## Methods

### Setting

This evaluation study takes place in four acute care hospitals servicing a single city, Calgary, Alberta, Canada, which has a referral population of approximately 1.7 million. Three of the four hospitals are academic hospitals operating a combined 56 adult medical-surgical ICU beds; the fourth is a nonacademic, community-based hospital operating 10 ICU beds. The annual ICU admission rate across the city approximates 3000 patients. In addition to the CIS hosting the e-transfer tool (ie, Sunrise Clinical Manager), all ICUs also use a dedicated provincial critical care CIS (ie, eCritical MetaVision) and clinical analytics system (ie, eCritical TRACER) that capture key demographic, clinical, health care service, and outcome data for all ICU patients [49]. ICUs are staffed by multidisciplinary teams; those in academic-based hospitals operate with a clinical fellow and 4 to 10 residents working under the supervision of an attending physician. One ICU has an NP. Critical care resident rotation blocks are 4 weeks in duration. The community-based ICU functions with an attending physician and 4 NPs.

### Study Design

This study uses a cluster-specific pre-post trial design with randomized and staggered implementation of the e-transfer tool across four hospitals.

### E-Transfer Tool Implementation

The e-transfer tool has been sequentially implemented into the four study hospitals at a new site every 2 months. This occurred between July 2018 and January 2019. The study biostatistician (AS), who was not involved with clinical practice in the ICUs, randomized the order of hospitals for implementation. Dictation remained available after implementation, but the ICU e-transfer tool was endorsed as the primary method to prepare ICU transfer summaries; as well, use of the tool was supported with strategies that have been successfully used in previous local initiatives, including in-person and web-based education, point-of-care support, and electronic audit and feedback [50].

### Participants

ICU patients from the four participating hospitals were eligible for inclusion in the study if the patient (1) was admitted to the ICU during the defined pre-post periods; (2) was 18 years of age or older; (3) had an ICU stay equal to, or longer than, 24 hours; and (4) was transferred from the ICU to an in-hospital PCU. Patient admission lists were extracted retrospectively by a data analyst with the critical care CIS repository (ie, eCritical TRACER). As the primary creators of most ICU transfer summaries [42], NPs and residents were invited to participate in a brief survey soliciting their experience creating transfer summaries.

### Data Collection

#### Overview

We set pre- and postimplementation data collection periods to extend for 6 months each, based on the staggered dates when the ICU e-transfer tool was implemented at each hospital. Patients transferred from the ICU prior to the intervention implementation date of their hospital are considered in the preimplementation period, while patients transferred from the ICU on or after the intervention implementation date of their hospital are considered in the postimplementation period.

**Figure 2:**
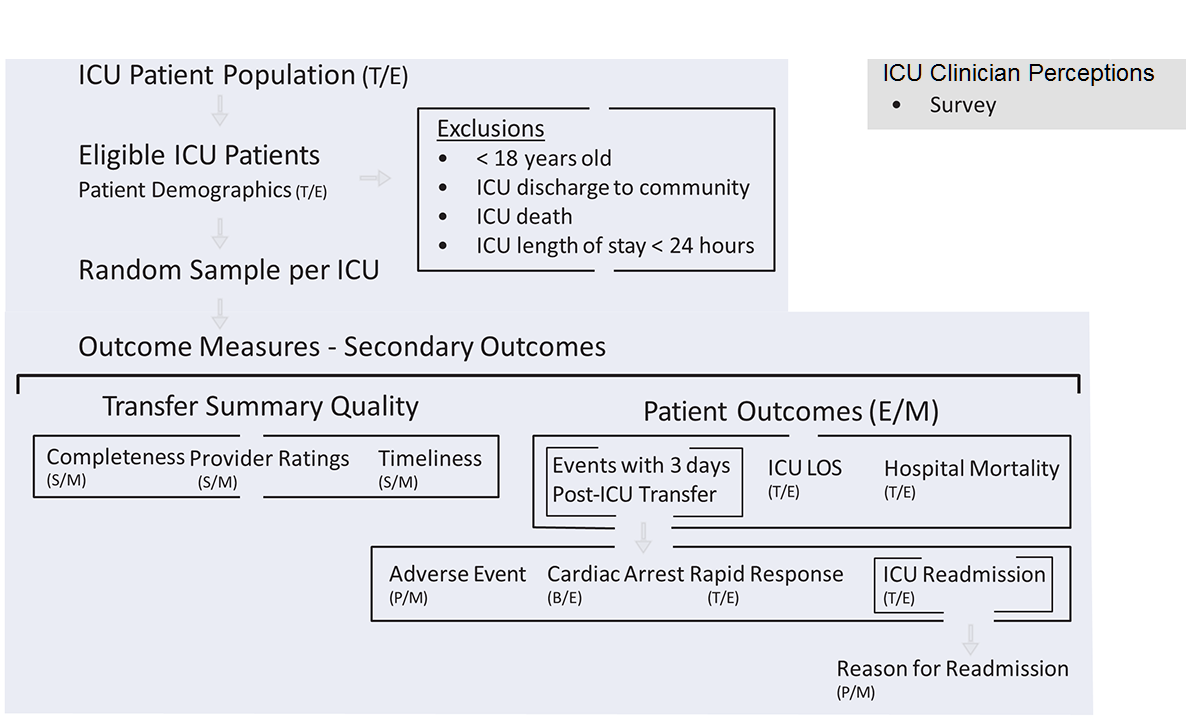
Data collection flow. B: critical care Code Blue database (data source); CIS: clinical information system; E: electronic extraction by CIS analyst (data collection method); ICU: intensive care unit; LOS: length of stay; M: manual extraction by study researcher (data collection method); P: paper chart (ie, medical doctor or nurse practitioner daily progress notes; data source); S: Hospital CIS (ie, Sunrise Clinical Manager; data source); T: critical care CIS analytics (ie, eCritical TRACER; data source).

Data collection involves (1) electronic extraction from provincial system repositories and a local critical care database, (2) manual abstraction from the patient’s electronic and paper medical record by trained researchers, and (3) manual rating of sampled transfer summaries by clinicians. Survey data of ICU clinician perspectives was collected pre- and postimplementation of the e-transfer tool. The flow of data collection is shown in Figure 2. Where feasible, we are deidentifying hospital name, dates, and clinician and patient identifiers from clinical documents (eg, transfer summaries and clinician progress notes) secured for manual data abstraction. All data will be encrypted and retained in a secured office.

#### Patient Demographics

Patient demographic data includes the following: age; sex; ICU and hospital admission and discharge dates, times, and locations; hospital mortality; comorbidities; ICU interventions (ie, intubation, ventilation, vasoactive medications, and dialysis); and severity of illness measures, including the Acute Physiology and Chronic Health Evaluation II (APACHE II) score [51], the Glasgow Coma Scale (GCS) score [52], and the Sequential Organ Failure Assessment (SOFA) score [53].

#### Outcome Measures

##### Overview

The primary outcome of interest is a binary composite measure of two conditions: information presence and availability (see Table 1). In the first condition, the presence of four essential information elements in the transfer summary—goals of care designation, diagnosis, list of active issues on transfer, and medications to continue on transfer—will be assessed and recorded as *yes* or *no*. All four elements must be present to be recorded as *yes*. In the second condition, the availability of the transfer summary to the accepting clinicians at the time of patient transfer from the ICU will be recorded as *yes* or *no*. Transfer summaries that meet these two conditions will be coded as *present*; those that do not will be coded as *absent*.

Secondary outcomes of interest fall into three main domains (see Table 1): (1) transfer summary quality (ie, completeness, timeliness, and clinician ratings), (2) patient outcomes (ie, post-ICU rapid response activation, cardiac arrest, adverse events, and ICU readmission), and (3) clinician perceptions. The rate of use of the e-transfer tool will also be measured by extracting the type of method—dictation or tool—used to prepare the medical summary for each patient transferred from the ICU during the study period.

**Table 1:** Evaluation outcome measures.

Domain and outcome		Outcome description
Primary outcome—binary (present or absent) composite measure of two conditions: information presence and availability (both conditions need to be met)		Presence of four essential information elements: Goals of care Diagnosis Problem issues on transfer Medications to continue on transfer Completed transfer summary available to patient care unit (PCU) at the time of patient’s transfer out of the intensive care unit (ICU)
**Transfer summary quality**		
	Overall completeness: proportion of eight requisite information elements present in transfer summary (%) and presence or absence of each essential information element in transfer summary	Summative score of the presence (score=1) of eight essential information elements—the four elements listed above and the following elements: Patient medical history Patient supportive needs (ie, venous thromboembolism prophylaxis, isolation, mobility, or nutrition) Patient attachments (ie, lines and tubes) Medication reconciliation
	Timeliness: availability of summary relative to the date and time of patient transfer (in hours)	Difference between the following: Date and time transfer summary was transcribed (ie, dictated summaries) or last edited (ie, electronic summaries) and Date and time of patient transfer from ICU
	Clinician ratings: clinician ratings of perceived general quality of transfer summary (median, IQR)	Rate five criteria on a 7-point Likert scale: Organization Completeness Pertinence Overall satisfaction Confidence that accepting team will understand patient care plan
**Patient outcomes**		
	Occurrence of negative patient outcomes within 3 days post–ICU transfer (%)	Patient events occurring within 3 days post-ICU: ICU readmission Adverse events Rapid response team activation Cardiac arrest
	Hospital and ICU total length of stay (in days)	Time between admission and discharge
	Mortality (in hours)	Time from ICU transfer to hospital mortality
ICU clinicians’ perceptions of their last transfer summary		Rate seven criteria on a 7-point Likert scale: Process: understood process to produce high-quality summary Workload: manageable to complete within routine ICU workflow Effectiveness: format able to communicate all relevant information clearly and logically Revisions: able to revise as new information becomes available Timely: able to complete at the time of patient transfer from ICU Satisfaction: produced a high-quality summary Confident that receiving PCU team will understand the patient care plan Length of time required to complete last transfer summary, including gathering all relevant information

##### Transfer Summary Quality

###### Completeness of Information

Trained researchers will manually abstract overall completeness of information in the summary. Completeness will be calculated as the sum of the individual binary scores (1=present; 0=absent) that the researchers will record for eight prospectively identified information elements prioritized as requisite from a list of 63 essential elements identified as important in ICU transfer summaries [43]. The eight information elements are as follows: goals of care designation, patient medical history, diagnosis, ICU active problem list, patient supportive care needs, patient attachments (ie, lines and tubes), active medications, and medication reconciliation. We designed a chart review form in REDCap (Research Electronic Data Capture) [54] (see Multimedia Appendix 1). As the researchers will need to access relevant clinical documents in study patients’ medical records, they will not be blinded to the study period or hospital.

###### Timeliness of Information

Timeliness of the summary is defined as the difference in hours between the date and time the patient transferred out of the ICU and the date and time the transfer summary was either transcribed, in the case of dictated documents, or last updated in the CIS, in the case of e-transfer tool documents.

###### Clinician Ratings

We will recruit ICU and PCU clinicians as volunteers to review and rate the general quality of a subsample of ICU transfer summaries randomly sampled from the larger pool of sampled summaries. Clinicians will use a 7-point scale to assess five criteria adapted from a previous study evaluating a similar tool [39,55]: organization (ie, presentation was logical and clear), completeness (ie, no information gaps or omissions), pertinence (ie, all content was relevant to patient care), overall satisfaction with the quality of the summary, and degree of confidence that the accepting clinician will understand the patient care plan after reading the transfer summary (see Table 1). Clinicians will be blinded to both the study period and hospital.

##### Patient Outcomes

Incidents of ICU readmissions and rapid response team activations occurring within 3 days of ICU transfer were extracted from the critical care system repository; cardiac events within 3 days of ICU transfer were extracted from the *Code Blue* database maintained within the Department of Critical Care Medicine (see Table 1). Patients who were readmitted to the ICU within 3 days of their first ICU transfer will be further evaluated by a clinician (see Figure 2) to determine if the reason for their readmission was related to a health issue documented in the transfer summary of their first ICU admission; this will be recorded as *yes*, *no*, or *unclear*.

Adverse events within 3 days of ICU transfer will be abstracted using a two-stage manual abstraction process based on the Institute for Healthcare Improvement Global Trigger Tool (GTT) method of chart review [56]. The GTT definition of an adverse event, as described on page 5 of the Institute for Healthcare Improvement white paper [56], is “any unintended physical injury resulting from or contributed to by medical care that requires additional monitoring, treatment or hospitalization, or that results in death.” In Stage 1, two trained researchers will independently review the daily clinician progress notes charted in each patient’s paper medical record 3 days post–ICU transfer. Using a list of 19 patient safety indicators, they will identify and record *yes*, *no*, or *unsure* for each incident of a suspected adverse event. The patient safety indicators are based on Southern and colleagues’ [57] list of 18 triggers adapted to a Canadian context with newer iterations of health coding data, with the addition of “patient falls.” In order to ensure good interrater reliability, Stage 1 reviewers will appraise a small sample of charts, compare results, and resolve any discrepancies before moving forward to evaluate the full sample. In Stage 2, any suspected adverse event recorded as *yes* or *unsure* during Stage 1 will be flagged for review by a third reviewer who will be a clinician. The clinician will review the notes and evaluate each suspected adverse event to confirm or reject the occurrence of the event using the GTT definition. In cases with a confirmed adverse event, the clinician reviewer will determine if the adverse event was preventable (ie, *yes*, *no*, or *unsure*), as well as designate the severity of the adverse event using the GTT categories of harm [56].

ICU and hospital length of stay will be captured using ICU and hospital admission and discharge dates and times. In-hospital mortality will be captured as the time from ICU discharge to hospital mortality, with censoring at hospital discharge for those who survived hospital.

##### Clinician Perceptions of Practice

To obtain ICU clinician feedback on preparing transfer summaries (see Table 1), we will analyze survey data collected pre- and postimplementation of the e-transfer tool. Our survey was adapted from a validated survey used to assess physician perceptions using a similar transfer tool [39,55]. We disseminated it via paper and online to ICU NPs and residents. The time between the two dissemination periods was over a year, making response bias unlikely. Participants were asked to rate their experience completing their last transfer summary on seven criteria: process (ie, understood what to include and how to accomplish this), workload (ie, completing was manageable within routine ICU workflow), effectiveness (ie, able to communicate all relevant information clearly and logically), revision (ie, able to easily edit and update the transfer summary with new information), timeliness (ie, able to complete by the time the patient is transferred from ICU), satisfaction (ie, summary was of high quality), and confidence that the accepting medical team will understand the patient care plan. Participants were also asked to estimate how long it took them, in minutes, to complete their last ICU transfer summary.

#### Sample Size Calculations

Sample size calculations were based on the cluster-specific pre-post study design. Based on our pilot [41,42], we calculated a required sample size of 144 pre- and 144 postimplementation ICU transfer summaries from each hospital to assess our primary outcome. This will be sufficient to detect an absolute difference in our primary outcome of 15% for each hospital with 82% power and an α value of 5% based on a baseline proportion of 20%; we observed a change in our pilot from 23% to 83%. A random sample of 24 ICU patients per hospital per month, over 6 months pre- and 6 months postimplementation, will facilitate secondary analyses, which accommodate the possibility of secular trends. The study biostatistician (AS) determined the random sample by assigning computer-generated random numbers to the complete list of patients transferred from each ICU within the study period, which was extracted by a data analyst with the critical care analytics system (see Figure 2). The study biostatistician was blinded to the method used to create the summary at the time of randomization.

To collect clinician ratings of the transfer summary quality, we calculated requiring 64 summaries preimplementation (ie, 16 per hospital × 4 hospitals = 64) and 64 summaries postimplementation (ie, 16 per hospital × 4 hospitals = 64), which will be sampled from aforementioned summaries, to detect an absolute difference in means as small as 0.5, assuming an SD of 1, with 80% power and an α value of 5%. The same patient cases will be used to assess for suspected post-ICU adverse events.

#### Data Analysis

Demographic characteristics pre- and postimplementation for each hospital will be described using medians with IQRs, means with SDs, and frequencies with percentages, as appropriate. The primary outcome will be compared within each hospital pre- and postimplementation using separate logistic regression models for each hospital, with adjustment for the following patient characteristics: age, sex, reason for ICU admission, status on ICU admission (ie, Charlson Comorbidity Index, APACHE II, GCS, and SOFA), therapies received while in ICU (ie, ventilation, vasoactive medications, intermittent hemodialysis, and continuous renal replacement therapy), status on transfer (ie, transfer delay time, transfer decision cancellations, and ICU occupancy), and ICU length of stay. Pooled analyses across all four hospitals will use mixed-effects logistic regression models with a fixed effect for intervention and a fixed effect for time in months, in order to model the underlying secular trend. A fixed effect for patient characteristics will also be used, as noted above, and random effects will be used for hospital and hospital by time to account for intracluster and interperiod correlation. In case of poor model fit or convergence issues due to a limited number of clusters, hospital-level analyses will be considered by aggregating the primary outcome over all summaries in each hospital during each month and using linear regression of the aggregated cluster-period proportions of complete and timely summaries with fixed effects for hospital and time in months. Secondary outcomes will be analyzed as described for the primary outcome, using within-hospital and pooled analyses. Wilcoxon rank-sum tests will be used to compare ICU and hospital length of stay, and log-rank tests will be used to compare time from ICU discharge to hospital mortality.

Open-ended comments collected through clinician surveys will be analyzed according to standard practices of qualitative textual analysis.

### Ethical Oversight and Trial Registration

The University of Calgary Conjoint Health Research Ethics Board reviewed this study (No. 17-2317) and granted a waiver of consent to collect retrospective data from relevant sections of patients’ paper medical records and EMRs. ICU clinicians who submit a survey will have implied their consent. Operational approvals and a data disclosure agreement was established with the provincial health custodian, Alberta Health Services. All protocol modifications will be reviewed by our research ethics board before being implemented. The trial was registered at ClinicalTrials.gov (NCT03590002).

## Results

Based on our study design, in fall 2019, the eCritical data analyst completed preliminary extraction of the list of patients transferred from the ICU within the 18-month range: February 12, 2018, to June 30, 2019. We have randomly sampled eligible patients from each ICU, restricting sampling to 6 months before and 6 months after the date the e-transfer tool was implemented in the hospital. Abstraction of primary and secondary outcomes is underway. We anticipate all data to be collected by early 2021, with data cleaning and analyses conducted and first results ready for publication in spring or summer 2021.

## Discussion

### Overview

The ICU e-transfer tool was designed to improve and standardize textual communication between clinicians during transitions in care from the ICU to other PCUs. The number of individuals who experience and recover from critical illness in their lifetime is steadily increasing. The proliferation of life-sustaining technologies has resulted in new challenges with transitions in care of newly vulnerable critically ill patients. We have documented significant gaps in continuity of care for ICU patients, one of the most clinically high-risk groups in the health care system [25,32]. The evidence-informed ICU e-transfer tool that we have developed and will evaluate can potentially optimize care across the health care continuum by mitigating communication errors and adverse events and contributing to improved experiences and outcomes for critically ill patients. Our evaluation will identify how the tool performs, what elements are effective, and what elements are ineffective and need to be refined or eliminated.

### Conclusions

This research will build a foundation for addressing an identified priority gap in patient care by rigorously evaluating a standardized electronic tool that will be adaptable to individual settings and scalable across health care jurisdictions. The study findings will add to the current literature on the effect of computerized tools on reducing communication breaks between the ICU and other PCUs during transitions in care and to ultimately improve patient safety.

## Appendix

Multimedia Appendix 1 Intensive care unit (ICU) transfer summary data abstraction form in REDCap (Research Electronic Data Capture).

Multimedia Appendix 2 Grant funding agency peer reviewer comments.

## Abbreviations

APACHE II: Acute Physiology and Chronic Health Evaluation II
CIS: clinical information system
DST: dictation, speech recognition, and transcription
EMR: electronic medical record
e-transfer: electronic transfer
GCS: Glasgow Coma Scale
GTT: Global Trigger Tool
ICU: intensive care unit
NP: nurse practitioner
PCU: patient care unit
REDCap: Research Electronic Data Capture
SOFA: Sequential Organ Failure Assessment

## References

[1] Li P, Stelfox HT, Ghali WA (2011). A prospective observational study of physician handoff for intensive-care-unit-to-ward patient transfers. Am J Med.

[2] Lin F, Chaboyer W, Wallis M (2009). A literature review of organisational, individual and teamwork factors contributing to the ICU discharge process. Aust Crit Care.

[3] Audit Commission (1999). Setting the Record Straight: A Review of the Progress in Health Records Services.

[4] The role of the ICU: What to expect. Canadian Critical Care Scociety.

[5] Prendergast TJ, Luce JM (1997). Increasing incidence of withholding and withdrawal of life support from the critically ill. Am J Respir Crit Care Med.

[6] Tonelli MR, Misak CJ (2010). Compromised autonomy and the seriously ill patient. Chest.

[7] Apker J, Mallak LA, Gibson SC (2007). Communicating in the "gray zone": Perceptions about emergency physician hospitalist handoffs and patient safety. Acad Emerg Med.

[8] Riesenberg LA, Leitzsch J, Massucci JL, Jaeger J, Rosenfeld JC, Patow C, Padmore JS, Karpovich KP (2009). Residents' and attending physicians' handoffs: A systematic review of the literature. Acad Med.

[9] Horwitz LI, Meredith T, Schuur JD, Shah NR, Kulkarni RG, Jenq GY (2009). Dropping the baton: A qualitative analysis of failures during the transition from emergency department to inpatient care. Ann Emerg Med.

[10] Pronovost P, Vohr E (2010). Safe Patients, Smart Hospitals: How One Doctor's Checklist Can Help Us Change Health Care from the Inside Out.

[11] Leith BA (1999). Patients' and family members' perceptions of transfer from intensive care. Heart Lung.

[12] Saarmann L (1993). Transfer out of critical care: Freedom or fear?. Crit Care Nurs Q.

[13] Odell M (2000). The patient's thoughts and feelings about their transfer from intensive care to the general ward. J Adv Nurs.

[14] Jacobs P, Noseworthy TW (1990). National estimates of intensive care utilization and costs: Canada and the United States. Crit Care Med.

[15] Needham DM, Bronskill SE, Calinawan JR, Sibbald WJ, Pronovost PJ, Laupacis A (2005). Projected incidence of mechanical ventilation in Ontario to 2026: Preparing for the aging baby boomers. Crit Care Med.

[16] Finfer S, Vincent J (2013). Critical care--An all-encompassing specialty. N Engl J Med.

[17] Stelfox HT, Soo A, Niven DJ, Fiest KM, Wunsch H, Rowan KM, Bagshaw SM (2018). Assessment of the safety of discharging select patients directly home from the intensive care unit: A multicenter population-based cohort study. JAMA Intern Med.

[18] Li P, Boyd JM, Ghali WA, Stelfox HT (2015). Stakeholder views regarding patient discharge from intensive care: Suboptimal quality and opportunities for improvement. Can Respir J.

[19] Bell CM, Brener SS, Gunraj N, Huo C, Bierman AS, Scales DC, Bajcar J, Zwarenstein M, Urbach DR (2011). Association of ICU or hospital admission with unintentional discontinuation of medications for chronic diseases. JAMA.

[20] Stelfox HT, Lane D, Boyd JM, Taylor S, Perrier L, Straus S, Zygun D, Zuege DJ (2015). A scoping review of patient discharge from intensive care: Opportunities and tools to improve care. Chest.

[21] Brooke J, Hasan N, Slark J, Sharma P (2012). Efficacy of information interventions in reducing transfer anxiety from a critical care setting to a general ward: A systematic review and meta-analysis. J Crit Care.

[22] Camiré Eric, Moyen E, Stelfox HT (2009). Medication errors in critical care: Risk factors, prevention and disclosure. CMAJ.

[23] Lyons PG, Arora VM, Farnan JM (2016). Adverse events and near-misses relating to intensive care unit-ward transfer: A qualitative analysis of resident perceptions. Ann Am Thorac Soc.

[24] Santhosh L, Lyons PG, Rojas JC, Ciesielski TM, Beach S, Farnan JM, Arora V (2019). Characterising ICU-ward handoffs at three academic medical centres: Process and perceptions. BMJ Qual Saf.

[25] de Grood C, Leigh JP, Bagshaw SM, Dodek PM, Fowler RA, Forster AJ, Boyd JM, Stelfox HT (2018). Patient, family and provider experiences with transfers from intensive care unit to hospital ward: A multicentre qualitative study. CMAJ.

[26] Santana MJ, Holroyd-Leduc J, Southern DA, Flemons WW, O'Beirne M, Hill MD, Forster AJ, White DE, Ghali WA, e-DCT Team (2017). A randomised controlled trial assessing the efficacy of an electronic discharge communication tool for preventing death or hospital readmission. BMJ Qual Saf.

[27] Mathioudakis A, Rousalova I, Gagnat AA, Saad N, Hardavella G (2016). How to keep good clinical records. Breathe (Sheff).

[28] Pilcher DV, Duke GJ, George C, Bailey MJ, Hart G (2007). After-hours discharge from intensive care increases the risk of readmission and death. Anaesth Intensive Care.

[29] Pronovost P, Weast B, Schwarz M, Wyskiel RM, Prow D, Milanovich SN, Berenholtz S, Dorman T, Lipsett P (2003). Medication reconciliation: A practical tool to reduce the risk of medication errors. J Crit Care.

[30] Graham AJ, Ocampo W, Southern DA, Falvi A, Sotiropoulos D, Wang B, Lonergan K, Vito B, Ghali WA, McFadden SDP (2019). Evaluation of an electronic health record structured discharge summary to provide real time adverse event reporting in thoracic surgery. BMJ Qual Saf.

[31] Stelfox HT, Leigh JP, Dodek PM, Turgeon AF, Forster AJ, Lamontagne F, Fowler RA, Soo A, Bagshaw SM (2017). A multi-center prospective cohort study of patient transfers from the intensive care unit to the hospital ward. Intensive Care Med.

[32] Brown KN, Leigh JP, Kamran H, Bagshaw SM, Fowler RA, Dodek PM, Turgeon AF, Forster AJ, Lamontagne F, Soo A, Stelfox HT (2018). Transfers from intensive care unit to hospital ward: A multicentre textual analysis of physician progress notes. Crit Care.

[33] Mehta RL, Baxendale B, Roth K, Caswell V, Le Jeune I, Hawkins J, Zedan H, Avery AJ (2017). Assessing the impact of the introduction of an electronic hospital discharge system on the completeness and timeliness of discharge communication: A before and after study. BMC Health Serv Res.

[34] Motamedi SM, Posadas-Calleja J, Straus S, Bates DW, Lorenzetti DL, Baylis B, Gilmour J, Kimpton S, Ghali WA (2011). The efficacy of computer-enabled discharge communication interventions: A systematic review. BMJ Qual Saf.

[35] O'Leary KJ, Liebovitz DM, Feinglass J, Liss DT, Evans DB, Kulkarni N, Landler MP, Baker DW (2009). Creating a better discharge summary: Improvement in quality and timeliness using an electronic discharge summary. J Hosp Med.

[36] Palma JP, Sharek PJ, Longhurst CA (2011). Impact of electronic medical record integration of a handoff tool on sign-out in a newborn intensive care unit. J Perinatol.

[37] Reinke CE, Kelz RR, Baillie CA, Norris A, Schmidt S, Wingate N, Myers JS (2014). Timeliness and quality of surgical discharge summaries after the implementation of an electronic format. Am J Surg.

[38] Kripalani S, LeFevre F, Phillips CO, Williams MV, Basaviah P, Baker DW (2007). Deficits in communication and information transfer between hospital-based and primary care physicians: Implications for patient safety and continuity of care. JAMA.

[39] de Grood C, Eso K, Santana MJ (2015). Physicians' experience adopting the electronic transfer of care communication tool: Barriers and opportunities. J Multidiscip Healthc.

[40] Granja C, Janssen W, Johansen MA (2018). Factors determining the success and failure of eHealth interventions: Systematic review of the literature. J Med Internet Res.

[41] Parsons Leigh J, Brundin-Mather R, Zjadewicz K, Soo A, Stelfox HT (2020). Improving transitions in care from intensive care units: Development and pilot testing of an electronic communication tool for healthcare providers. J Crit Care.

[42] Boyd JM, Roberts DJ, Parsons Leigh J, Stelfox HT (2018). Administrator perspectives on ICU-to-ward transfers and content contained in existing transfer tools: A cross-sectional survey. J Gen Intern Med.

[43] de Grood C, Job McIntosh C, Boyd JM, Zjadewicz K, Parsons Leigh J, Stelfox HT, ICU Transfer Summary Consensus Panel (2019). Identifying essential elements to include in intensive care unit to hospital ward transfer summaries: A consensus methodology. J Crit Care.

[44] Donabedian A (1966). Evaluating the quality of medical care. Milbank Mem Fund Q.

[45] Maher L, Gustafson D, Evans A (2010). NHS Sustainability Model and Guide.

[46] Stelfox HT, Brundin-Mather R, Soo A, Parsons Leigh J, Niven DJ, Fiest KM, Doig CJ, Zuege DJ, Kushner B, Clement F, Straus SE, Cook DJ, Bagshaw SM, Sauro KM (2019). A multicentre controlled pre-post trial of an implementation science intervention to improve venous thromboembolism prophylaxis in critically ill patients. Intensive Care Med.

[47] Stelfox HT, Bobranska-Artiuch B, Nathens A, Straus SE (2010). Quality indicators for evaluating trauma care: A scoping review. Arch Surg.

[48] Doyle C, Howe C, Woodcock T, Myron R, Phekoo K, McNicholas C, Saffer J, Bell D (2013). Making change last: Applying the NHS institute for innovation and improvement sustainability model to healthcare improvement. Implement Sci.

[49] Brundin-Mather R, Soo A, Zuege DJ, Niven DJ, Fiest K, Doig CJ, Zygun D, Boyd JM, Parsons Leigh J, Bagshaw SM, Stelfox HT (2018). Secondary EMR data for quality improvement and research: A comparison of manual and electronic data collection from an integrated critical care electronic medical record system. J Crit Care.

[50] Sauro KM, Brundin-Mather R, Parsons Leigh J, Niven DJ, Kushner B, Soo A, Cook DJ, Straus S, Doig CJ, Bagshaw S, Stelfox HT (2019). Improving the adoption of optimal venous thromboembolism prophylaxis in critically ill patients: A process evaluation of a complex quality improvement initiative. J Crit Care.

[51] Knaus WA, Draper EA, Wagner DP, Zimmerman JE (1985). APACHE II: A severity of disease classification system. Crit Care Med.

[52] Teasdale G, Jennett B (1974). Assessment of coma and impaired consciousness. A practical scale. Lancet.

[53] Vincent JL, Moreno R, Takala J, Willatts S, De Mendonça A, Bruining H, Reinhart CK, Suter PM, Thijs LG (1996). The SOFA (Sepsis-related Organ Failure Assessment) score to describe organ dysfunction/failure. On behalf of the Working Group on Sepsis-Related Problems of the European Society of Intensive Care Medicine. Intensive Care Med.

[54] Harris PA, Taylor R, Minor BL, Elliott V, Fernandez M, O'Neal L, McLeod L, Delacqua G, Delacqua F, Kirby J, Duda SN, REDCap Consortium (2019). The REDCap consortium: Building an international community of software platform partners. J Biomed Inform.

[55] Santana MJ, Holroyd-Leduc J, Flemons WW, O'Beirne M, White D, Clayden N, Forster AJ, Ghali WA (2014). The seamless transfer of care: A pilot study assessing the usability of an electronic transfer of care communication tool. Am J Med Qual.

[56] Griffin FA, Resar RK (2009). IHI Global Trigger Tool for Measuring Adverse Events (Second Edition). IHI Innovation Series white paper.

[57] Southern DA, Burnand B, Droesler SE, Flemons W, Forster AJ, Gurevich Y, Harrison J, Quan H, Pincus HA, Romano PS, Sundararajan V, Kostanjsek N, Ghali WA (2017). Deriving ICD-10 codes for patient safety indicators for large-scale surveillance using administrative hospital data. Med Care.

