# Supplementary material for: Effectiveness of an Electronic Communication Tool on Transitions in Care From the Intensive Care Unit: Protocol for a Cluster-Specific Pre-Post Trial
Source: JMIR Res Protoc. 2021 Jan 8;10(1):e18675. doi: 10.2196/18675 (PMC7822720; doi:10.2196/18675)
Supplement: Multimedia Appendix 1 [file resprot_v10i1e18675_app1.pdf]

Appendix 1. ICU Transfer Summary Data Abstraction Form in REDCap

Chart Demographics

Adding new Record ID **test**

Record ID test

Data Abstractor
* must provide value

- RBM
- LWB
- JH
- Other
- MA

reset

Abstraction Date

Now Y-M-D H:M

Hospital
Patient Location
* must provide value

- FMC
- RGH
- PLC
- SHC

reset

Type of Summary Document
ICU Summaries completed by SCM Note will correspond directly to the choices to the right, under the heading "Document Topic"; dictated summaries may not be immediately obvious if transfer or discharge.
* must provide value

- Transfer
- Discharge
- Deceased
- Entered in Error
- Not certain based on document content

reset

Method Used to Prepare ICU Summary
* must provide value

- Dictation
- SCM Note
- Unknown

reset

Primary Author of ICU Summary
Go to 'Status' tab in Summary
If Summary does not say dictated by but is signed by a Nurse Practitioner, we will assume that it also was dictated by the NP. Therefore, the NP is the primary author.
* must provide value

- Resident
- Fellow
- NP
- Attending
- Other
- Absent

reset

Number of times ICU summary was edited
Go to 'Status' tab in Summary > Count entries.
*** Do not count the MD/NP finalizing the document in your count.***
-
Dictated summaries will likely be 1 but in the situation where a dictated summary shows multiple edits, be cautious. Additions of addenda do NOT count as edits.
Be critical when looking at the dictation and revision times to help identify the situation.

Number of authors that edited ICU summary
Go to 'Status' tab in Summary > Count Authors
*** Do not count the MD/NP finalizing the document in your count.***

Who contributed to the ICU Summary, in addition to the primary author?
* Can include the MD/NP who may have finalized the summary.*

- ☐ None (only one author)
- ☐ Resident
- ☐ Fellow
- ☐ NP
- ☐ Attending
- ☐ Clinical Clerk
- ☐ Other

Abstraction Notes

Expand

Completeness Of Icu Summary Content

Adding new Record ID **test**

Record ID test

Completeness of ICU Summary

	Content Present	Content Absent	Unsure
**Goals of Care**	○	○	○
**Patient Medical History**<br>**This includes any reference to past events and circumstances that may be relevant to a patient's current state of health. This may include, but not limited to, an account of past diseases, injuries, treatments, chronic issues, surgeries, family history, social history and tobacco use.**<br>**It may be described as a *Profile*.**<br>**This may include qualifying statements like "previously healthy".**<br>**This is not History of Presenting Illness.**	○	○	○
**Diagnosis**<br>**Hospital and/or Primary Diagnosis is acceptable.**<br>***may be free texted as "Most Responsible Diagnosis" (MRD)***	○	○	○

reset

reset

ICU Problem List

ICU problem list should note and/or describe active medical issues on transfer out of ICU - i.e., issues/events OR status of patient on transfer. There should be some indication of the current status of patient health issues noted described typically within Course in ICU (are they active, resolved, follow-up, etc). ICU problem list may be listed under an independent heading or embedded with descriptive text.

○ ○ ○

reset

List of Active Medications

List of medications patient is currently on

○ ○ ○

reset

Medication Reconciliation

Score 'present' if evidence to indicate that a comprehensive review of all patients medications (hospital & home) was done and reconciled (e.g., comments/rationale for discontinuation, holds, or changes to home meds, dose, route) so new providers have accurate list to compare with new orders. A statement that No medication changes is also evidence of Med Rec.

Lists of home meds and active meds is not sufficient.

○ ○ ○

reset

Send Copies to

For dictated summaries, look for providers who are CC'ed.

○ ○ ○

reset

Patient Needs (Supportive Care)

Check box if you see content related to the respective item.

*** If author notes patient is receiving VTE treatment (e.g., IV Heparin, Argrotoban), can check DVT/VTE prophylaxis**

*** Pulmonary embolisms (PE) are a type of VTE**

- ☐ DVT/VTE prophylaxis *
- ☐ Isolation
- ☐ Mobility limitations
- ☐ Nutrition (includes swallowing difficulties)
- ☐ Other
- ☐ N/A (only check if author *explicitly* indicated patient had No patient needs.)
- ☐ Content Absent (author has omitted information pertinent to this field)

Patient Needs Field 0 View equation

Patient Attachments

Check box if you see content related to the respective item.

- ☐ Central line
- ☐ Chest tube
- ☐ CPAP/BiPAP
- ☐ Dialysis access
- ☐ Surgical drain
- ☐ Feeding tube
- ☐ Foley
- ☐ Rectal tube
- ☐ Tracheostomy
- ☐ Other
- ☐ NONE (only check if author has *explicitly* indicated No attachments.)
- ☐ Content Absent (author has omitted information pertinent to this field)

Patient Attachments Field 0 View equation

Abstraction Notes
