# Supplementary material for: Effectiveness of an Electronic Communication Tool on Transitions in Care From the Intensive Care Unit: Protocol for a Cluster-Specific Pre-Post Trial
Source: JMIR Res Protoc. 2021 Jan 8;10(1):e18675. doi: 10.2196/18675 (PMC7822720; doi:10.2196/18675)
Supplement: Multimedia Appendix 2 [file resprot_v10i1e18675_app2.pdf]

Review Type/Type d'évaluation: Committee Member 1/Membre de comité 1
Name of Applicant/Nom du chercheur: Parsons Leigh, Jeanna Joanne
Application No./Numéro de demande: 420324
Agency/Agence: CIHR/IRSC
Competition/Concours: 2019-02-05 Operating Grant: Transitions in Care - Evaluation Grants/Subvention de fonctionnement : Transitions dans les soins - subventions d'évaluation
Committee/Comité: Operating Grant: Transitions in Care – Evaluation Grants/Subvention de fonctionnement : Transitions dans les soins – subventions d'évaluation
Title/Titre: Improving Transitions in Care from ICU: Evaluation of an Electronic Communication Tool

---

Assessment/Évaluation:

Research Question
The rationale for the proposed research is nicely situated within the literature. The applicants note several statistics related to transitions in care between the ICU and hospital wards that provide a strong motivation for improving the stated limitations in the current system to support these transitions. Additionally, the background work that informed the development of the existing evidence-based ICU Transfer Tool that has been implemented in four ICU's in Calgary, Alberta is admirable. The applicants propose evaluating the tool for effective communication between medical teams, patient- and provider-reported outcomes, and healthcare utilization, and then disseminating the findings nationally. The project responds nicely to the funding call objectives and aligns with the TiC focus areas.

Research Approach
The first phase of the project, evaluation, extends the team's previous work developing the ICU Transfer Tool and testing the tool in a small feasibility pilot in one site. The proposed study will utilize a non-randomized cluster pre-post stepped wedge RCT. The intervention arm will deliver the ICU Transfer Tool, and the control arm will receive standard care (dictated ICU transitions). Adult-only patients will be cluster randomized by site, which is appropriate for this type of intervention and to allow the study team to support each trial. The outcomes are somewhat primitive (e.g., the patient demographics and patient-oriented outcomes) or not grounded nicely in the literature (e.g., ICU transfer summary quality, satisfaction with practice change). The proposed analysis methodology does not seem consistent with best practices for stepped wedge study designs (e.g., GLM, GEE); rather the applicants propose treating each site as a separate trial.
The second phase of the project focuses on developing a KT Kit that will support future roll-out of the tool across Alberta. The team proposes 6-12 focus groups to elicit multi-dimensional perspectives from stakeholders. These focus groups will inform the development of a survey tool that will be administered by phone to 140 stakeholders. The methodology outlined in this phase is not strong. For example, the focus groups will be analysed "according to standard practices of qualitative research" is inappropriately presented. Sex and gender are not adequately accounted for. Indigenous cultural understandings are not addressed.

Applicants
The team is appropriate for the proposed study, with previous experience with this population, with the intervention, and with the study sites. The team will also have access to the necessary resources to successfully execute the project. Knowledge users are appropriately engaged in the KT phase of the study, but not involved directly in the evaluation.

Feasibility
The budget is appropriate and makes good use of graduate students and other trainees. There is notably minimal allocation of funding for travel and dissemination beyond support for a conference presentation in

**Review Type/Type d'évaluation:**	Committee Member 1/Membre de comité 1
**Name of Applicant/Nom du chercheur:**	Parsons Leigh, Jeanna Joanne
**Application No./Numéro de demande:**	420324
**Agency/Agence:**	CIHR/IRSC
**Competition/Concours:**	2019-02-05 Operating Grant: Transitions in Care - Evaluation Grants/Subvention de fonctionnement : Transitions dans les soins - subventions d’évaluation
**Committee/Comité:**	Operating Grant: Transitions in Care – Evaluation Grants/Subvention de fonctionnement : Transitions dans les soins – subventions d’évaluation
**Title/Titre:**	Improving Transitions in Care from ICU: Evaluation of an Electronic Communication Tool

Assessment/Évaluation:

year two. The environment is suitable to support the project, and I do not see any issues with the feasibility of completing the project according to the proposed timeline.

Impact of Research
The dissemination plan for the project is good with respect to engaging stakeholders and preparing for provincial/national scale. However, the dissemination plan academically to the scientific community is weak. The proposed work, if successful, may have a significant impact on transitions in care provincially and nationally as well.

Review Type/Type d'évaluation: Committee Member 2/Membre de comité 2
Name of Applicant/Nom du chercheur: Parsons Leigh, Jeanna Joanne
Application No./Numéro de demande: 420324
Agency/Agence: CIHR/IRSC
Competition/Concours: 2019-02-05 Operating Grant: Transitions in Care - Evaluation Grants/Subvention de fonctionnement : Transitions dans les soins - subventions d'évaluation
Committee/Comité: Operating Grant: Transitions in Care – Evaluation Grants/Subvention de fonctionnement : Transitions dans les soins – subventions d'évaluation
Title/Titre: Improving Transitions in Care from ICU: Evaluation of an Electronic Communication Tool

---

Assessment/Évaluation:

SUMMARY
The proposed research is designed to address a key gap in the transition of patients from the ICU to hospital ward or to home. With the support of key stakeholders and previous funding obtained from CIHR and the Canadian Frailty Network, the team has developed a nonproprietary electronic ICU Transfer Tool. On the basis of pilot testing that has been completed with this tool, there are 2 phases of research that are proposed. In Phase 1, the team will conduct an adapted non-blinded pre-post randomized cluster (hospital-level) stepped-wedge design to evaluate the quality of ICU transfer. Atrained auditor will review the presence /absence of 8 medical information fields on the transfer record and score these on 5 dimensions: organization, completeness, pertinence, effectiveness and understanding of the patient care plan. A composite measure of completeness and timeliness will be derived as the outcome variable. In Phase 2, a KT Tool Kit will be developed to facilitate dissemination of this ICU transfer tool across Alberta. This phase will follow input from focus groups with key stakeholders. Surveys will also be conducted using purposive sampling. Findings from Phases 1 and 2 will be disseminated to key stakeholders to support subsequent roll out in Alberta and beyond.

Scientific rationale. The literature review provides a clear rationale as to the need for this transition in care proposal. It highlights that there is a gap in the transition from ICU to the hospital ward or home. It notes that impaired communication is a “root cause” for this gap which results in preventable medical errors, redundant testing, readmissions to the ICU or hospital, and dissatisfaction with quality of care. The team has consulted with key stakeholders and secured previous funding. Moreover, pilot testing has been completed. Positive results were reported for the benefit of the electronic ICU Transfer Tool vs. a “dictated cohort”, which is the current standard of care—although this pilot study was not referenced as a presentation or publication.

RESEARCH APPROACH
Strengths.
This proposal was a pleasure to read. The literature review presents evidence to support the need for this proposed research in a succinct manner, while effectively demonstrating the need for this proposed transition in care research. The research design for Phase 1 and 2 is clearly presented, appropriate, and well documented with regard to objectives, procedures, design models, outcomes, and analyses. Additional details of the procedures that stand out include the fact that ICU and frontline providers blinded to the study will rate the quality of ICU transfer summaries, and their satisfaction with documentation practices (Data Collection/Sources section). In addition, the inclusion of all patients admitted to the ICU for this study will ensure the generalizability of the findings—which is important for scaling this procedure to other hospitals outside of Alberta. The feasibility of this project is extremely well documented in terms of the background range of inter-professional and multidisciplinary expertise of the team, previous engagement with stakeholders, completion of previous pilot research, and the KT plan for disseminating findings.

Review Type/Type d'évaluation: Committee Member 2/Membre de comité 2
Name of Applicant/Nom du chercheur: Parsons Leigh, Jeanna Joanne
Application No./Numéro de demande: 420324
Agency/Agence: CIHR/IRSC
Competition/Concours: 2019-02-05 Operating Grant: Transitions in Care - Evaluation Grants/Subvention de fonctionnement : Transitions dans les soins - subventions d'évaluation
Committee/Comité: Operating Grant: Transitions in Care – Evaluation Grants/Subvention de fonctionnement : Transitions dans les soins – subventions d'évaluation
Title/Titre: Improving Transitions in Care from ICU: Evaluation of an Electronic Communication Tool

---

Assessment/Évaluation:

Weaknesses.
Outcomes: The efficacy of the ICU Transfer Tool will be evaluated using a "trained auditor who will review the presence/absence of 8 medical information fields on the transfer record and score these on 5 dimensions: organization, completeness, pertinence, effectiveness and understanding of the patient care plan. Given the importance of this evaluation for the Phase 1 project, the strength of this study would have been increased with evaluations being conducted by at least 2 independent auditors, where their ratings could be reviewed for level of agreement, and where any differences in ratings could be resolved by consensus involving a 3rd party. This would improve the actual and perceived objectivity of this assessment, while providing information on the reliability of the ratings about how the device is used in practice.

The proposal notes that the outcome will be derived from 8 medical information fields on the transfer record and scored on 5 dimensions: organization, completeness, pertinence, effectiveness and understanding of the patient care plan. A composite measure of completeness and timeliness will be derived and then recoded into a binary variable to reflect the presence/absence of four key information fields: goals of care, diagnosis, active problem list and medications to continue. This assessment will also reflect whether the record is available at time of transfer from the ICU. This process represents several steps in the merging and recoding of outcome data. This section would have been strengthened with a clear definition of the criteria by which each of the final 4 information fields would receive a positive score using the binary code. Further, I wondered whether a scoring index on a continuous (vs binary) scale would be more informative about clinical practice.

It is also noted that the research team will engage stakeholders and opinion leaders at each of the 4 participating hospitals (unit managers, clinicians and nursing staff) to facilitate adoption of the ICU Transfer Tool by physicians, nurse practitioners, and nurses: "…critical care leaders…will encourage clinicians to use the ICU Transfer Tool as the default system." Adherence of staff to use this tool is a potential issue – which (in fairness) is addressed in this proposal. Nevertheless, in preparation for scaling the adoption of this electronic tool, it would seem advisable to collect and analyze usability data from the Phase 1 study. This would provide requisite information on the rate at which the ICU Transfer Tool was utilized by staff.

The analyses for this investigation are not well described, which was surprising given the pilot work that has already gone into this initiative. It was surprising to see that the outcomes for Phase 1 of this research will be evaluated using univariable analyses: chi-squared test, t-tests, and Wilcoxon rank sum test. The data analysis section states that sensitivity analyses will adjust for patient and hospital characteristics using logistic or linear regress models, but how this will be conducted remains vague. This section would have been markedly improved if the primary outcome analysis was described in terms of a multivariable analysis (e.g. the regression models) with an indication how confounding factors (moderators or mediators) would be

Review Type/Type d'évaluation: Committee Member 2/Membre de comité 2
Name of Applicant/Nom du chercheur: Parsons Leigh, Jeanna Joanne
Application No./Numéro de demande: 420324
Agency/Agence: CIHR/IRSC
Competition/Concours: 2019-02-05 Operating Grant: Transitions in Care - Evaluation Grants/Subvention de fonctionnement : Transitions dans les soins - subventions d'évaluation
Committee/Comité: Operating Grant: Transitions in Care – Evaluation Grants/Subvention de fonctionnement : Transitions dans les soins – subventions d'évaluation
Title/Titre: Improving Transitions in Care from ICU: Evaluation of an Electronic Communication Tool

---

Assessment/Évaluation:

incorporated into the model. Further, given the complexity of the ICU population, it is likely that the primary outcome will be analysed using several regression models. A brief summary of the key models to be used would have been helpful.

Applicants:
The PI (Dr. Parsons Leigh) is an *early* career investigator with expertise in sociology, new knowledge acquisition and collaboration, critical care, and knowledge translation.Over the past 7 years, peer reviewed funding for which she has been a Co-Applicant is approximately $4,630,000. Within this total, she has had success as a PI, with approximately $530,000. Over the past 7 years, Dr. Parsons Leigh has 22 peer-reviewed publications, with 8 as first or last author. She is *currently*Supervisor or Academic Advisor for 2 MSc students. Other members of the team have demonstrated expertise in patient and family engagement, critical care medicine, nursing, palliative care, geriatrics, primary care, rehabilitation, health economics, implementation science, and biostatistics. They hold senior positions with key stakeholder groups and they have an impressive record of publications (e.g. NEJM, JAMA, Lancet) for studies conducted at the local, national and international level. They report approximately $119 million in funding as PI's.

Budget
No issues.

Impact of Research and KT.
The knowledge translation plan is clearly presented. There is strong support from key stakeholders (Alberta Health Services, Canadian Critical Care Trials Group/Society and participating hospitals). KT plans for the ICU transfer Tool include making it available via open access as a non-proprietary, standardized, evidence-informed electronic ICU suitable for national and international application. Further, the team will engage knowledge users and knowledge brokers through professional and organizational methods (e.g. newsletters), and they will host a national forum ontransitions of patient care to link researchers with knowledge users. The ultimate goal is to influence the standard of practice by rolling out this ICU Transfer Tool and the KT Tool Kit to national and international settings.

**Review Type/Type d'évaluation:**	Committee Member 3/Membre de comité 3
**Name of Applicant/Nom du chercheur:**	Parsons Leigh, Jeanna Joanne
**Application No./Numéro de demande:**	420324
**Agency/Agence:**	CIHR/IRSC
**Competition/Concours:**	2019-02-05 Operating Grant: Transitions in Care - Evaluation Grants/Subvention de fonctionnement : Transitions dans les soins - subventions d'évaluation
**Committee/Comité:**	Operating Grant: Transitions in Care – Evaluation Grants/Subvention de fonctionnement : Transitions dans les soins – subventions d'évaluation
**Title/Titre:**	Improving Transitions in Care from ICU: Evaluation of an Electronic Communication Tool

Assessment/Évaluation:

Competition:CIHR Operating/Evaluation Grant: Transitions In Care 2019

Project Application Title: OG420324 – Improving Transitions in Care from ICU: Evaluation of an Electronic Communication Tool

1. Research Question
a. Research question(s) is/are clearly stated. **- YES**
b. Strong scientific rationale for pursuing the proposed evaluation. **– STRONG**
c. Extent to which the research project responds to the objectives of the funding opportunity. **- HIGH**

2. Research Approach
a. Strength of the research approach and justification for the proposed methods/strategies that is supported by available evidence and/or literature. **- STRONG**
b. Appropriateness and rigor of the proposed study design to address the research question(s). **- HIGH**
c. Appropriate incorporation and justification of sex as a biological variable and/or gender as a social determinant of health where applicable. **- YES**
d. Appropriate incorporation of Indigenous culturally relevant theoretical and conceptual frameworks, and Indigenous culturally appropriate research protocols, including Indigenous methodologies where applicable. **- YES**

3. Applicants
a. Strength of the applicants, taking into consideration evidence that there is the appropriate expertise, influence, resources and stakeholders who are appropriately and meaningfully involved. **- HIGH**
b. Appropriate engagement of the knowledge user(s) responsible for, or involved in decision-making of, the activity being evaluated. **- HIGH**

4. Feasibility
a. Appropriateness of the budget and the justification for the amount requested, including the required budget for knowledge translation and dissemination activities. **- TBD**
b. Suitability of the environment, including availability and accessibility of personnel and tools, to conduct the proposed activities. **- HIGH**
c. Probability that the project objectives will be met within the proposed timeline. **- TBD**

5. Impact of Research
a. Strength of the dissemination plan. **- STRONG**
b. Potential of the proposal to advance knowledge and produce high-quality evidence to inform actionable

**Review Type/Type d'évaluation:**	Committee Member 3/Membre de comité 3
**Name of Applicant/Nom du chercheur:**	Parsons Leigh, Jeanna Joanne
**Application No./Numéro de demande:**	420324
**Agency/Agence:**	CIHR/IRSC
**Competition/Concours:**	2019-02-05 Operating Grant: Transitions in Care - Evaluation Grants/Subvention de fonctionnement : Transitions dans les soins - subventions d'évaluation
**Committee/Comité:**	Operating Grant: Transitions in Care – Evaluation Grants/Subvention de fonctionnement : Transitions dans les soins – subventions d'évaluation
**Title/Titre:**	Improving Transitions in Care from ICU: Evaluation of an Electronic Communication Tool

Assessment/Évaluation:

health system changes to improve care transitions. **- STRONG**

NOTES/COMMENTS: A comprehensively crafted and broadly supported innovation that shows promise to help ensure better continuity of team-based care and collaborative communication for discharge-planned patients transitioning out of ICU as a survivable setting. ICU-induced psychosis awareness and ongoing integration of overall mental health care are vital considerations. What role will de-prescribing of medications also play in patient discharge and follow-up?

Review Type/Type d'évaluation: Committee Member 4/Membre de comité 4
Name of Applicant/Nom du chercheur: Parsons Leigh, Jeanna Joanne
Application No./Numéro de demande: 420324
Agency/Agence: CIHR/IRSC
Competition/Concours: 2019-02-05 Operating Grant: Transitions in Care - Evaluation Grants/Subvention de fonctionnement : Transitions dans les soins - subventions d’évaluation
Committee/Comité: Operating Grant: Transitions in Care – Evaluation Grants/Subvention de fonctionnement : Transitions dans les soins – subventions d’évaluation
Title/Titre: Improving Transitions in Care from ICU: Evaluation of an Electronic Communication Tool

---

Assessment/Évaluation:

Research Questions: The applicant proposes a clearly formulated research question related to the ICU transfer tool evaluation. There is strong evidence supporting the pursuit of this particular question which is relevant in the context of the funding application. The 2nd topic is not a research question but rather the proposed solution to be developed based on findings in phase 1.
Research Approach: for the phase 1 of the proposed project, the proposed methodology is appropriate and supports the objectives of the research. Approach to data collection and available outcomes for evaluation are feasible. The significance threshold for decision makers appears rather modest (15% absolute increase). Phase 2 of the project consists of the development of KT tool kit to support spread and scale - not a research proposal but the development of the proposed scalable solution enabled by results in phase 1. For the stated purpose of phase 2, the proposed approach is reasonable and achievable. However it is not clear what happens if the pre-defined significance threshold is not achieved. The phase 2 should be conditioned by phase 1 outcomes. Potential confounders of phase 1 results as well as other limitations that can be created by e.g. center selection bias are not described.
Applicants: qualified team with appropriate representation of key stakeholders for the proposed transition in care
Feasibility: proposed budget is reasonable vs proposed activities, environment for research is appropriate and ensures access to key data required for the research to be completed, risks identified and risk mitigation strategies proposed, timelines are achievable. Phase 2 of the project should be conditioned by outcomes of phase 1.
Impact of Research: publication dissemination plan beyond communication to key stakeholders and development of the KT tool in phase 2 is not detailed. However, the applicant is committed to develop and roll-out, based on findings in phase 1, the KT tool at provincial and national level with the clear aim to create actionable health system changes to improve care transitions for ICU admitted patients in Canada.

Review Type/Type d'évaluation: SO Notes /Notes de l'agent scientifique
Name of Applicant/Nom du chercheur: Parsons Leigh, Jeanna Joanne
Application No./Numéro de demande: 420324
Agency/Agence: CIHR/IRSC
Competition/Concours: 2019-02-05 Operating Grant: Transitions in Care - Evaluation Grants/Subvention de fonctionnement : Transitions dans les soins - subventions d’évaluation
Committee/Comité: Operating Grant: Transitions in Care – Evaluation Grants/Subvention de fonctionnement : Transitions dans les soins – subventions d’évaluation
Title/Titre: Improving Transitions in Care from ICU: Evaluation of an Electronic Communication Tool

Assessment/Évaluation:

This proposal evaluates an electronic tool aimed at improving communication between ICU and other medical staff for individuals being discharged from the ICU. Strengths noted by the committee included the rationale for the proposed work, the previous pilot work that this proposal builds on, the good fit with the CIHR call, the engagement of KUs in the dissemination plan, and the theoretically informed and evidence-based approach. The fact that this tool is non-proprietary and the framing of the ICU as a survivable environment were also noted as pluses.

Committee discussion included whether more nuanced outcomes measures should be considered, whether treating the three sites as separate RCTs was consistent with a stepped-wedge design, the need for a more fulsome description of how the progress from focus group to surveys would occur, and the need for KUs to be involved in the evaluation phase as well. The value of adding another trained auditor was discussed as was the value of having a template to support the extensive data reduction process described. More information on the adherence of staff to using the tool would have been helpful. How potential mental health impacts of the ICU would be captured and addressed and also how medication discontinuation post-discharge might be handled were also raised.

Finally, the strength and thoroughness of the phase 1 description was not matched by the phase 2 description. For example, how the Donabedian framework or the Triple Aim are actually operationalized could use more description. Also, more clarity on how phase 1 would influence phase 2 would have been helpful.

Attention to these concerns would strengthen this already strong proposal.
